# A cluster randomized controlled trial comparing the effectiveness of two school-based interventions for autistic youth with anxiety

**DOI:** 10.1186/s12888-023-05441-0

**Published:** 2024-01-02

**Authors:** Katherine Pickard, Brenna Maddox, Richard Boles, Judy Reaven

**Affiliations:** 1 Emory School of Medicine Department of Pediatrics, Division of Autism and Related Disabilities, 1920 Briarcliff Road, Atlanta, GA 30329 USA; 2 University of North Carolina, Chapel Hill, North Carolina USA; 3 University of Colorado Anschutz Medical Campus, JFK Partners, Aurora, CO USA

**Keywords:** Autism, Anxiety, Schools, Cognitive behavioral therapy (CBT), Implementation, Patient-centered outcomes

## Abstract

**Background:**

Recent systematic reviews have indicated that cognitive behavioral therapy (CBT) is effective in reducing anxiety symptoms for autistic and non-autistic children. However, the vast majority of CBT research for autistic youth has been implemented within university settings and primarily by mental health providers. Schools hold great promise to equitably manage the mental health symptoms of autistic youth. Although preliminary research evaluating CBT within schools has been promising, CBT has not yet been compared to another readily available school mental health program. The goal of this protocol paper is to describe a multi-site study comparing two school-based interventions, Facing Your Fears-School Based (FYF-SB) and Zones of Regulation (ZOR) via a cluster randomized controlled type 1 hybrid effectiveness-implementation trial to determine which of the two interventions will best support autistic youth with anxiety in schools.

**Methods:**

Up to 100 elementary and middle schools will be randomized into FYF-SB or ZOR. Once schools are randomized, a minimum of two interdisciplinary school providers at each school will be trained to deliver either FYF-SB or ZOR over the course of 12 weeks to groups of 2–5 autistic students ages 8–14 years*.* Over the course of two years, a total of 200 autistic students will receive either ZOR or FYF-SB. The primary outcome of this trial is child anxiety, as rated by masked evaluators and via caregiver- and student-report, which will be measured at baseline, post-treatment, and 6-month follow-up. Semi-structured interviews will also be conducted with a purposive sample of students, caregivers, and school providers to understand the acceptability, appropriateness, and feasibility of either ZOR or FYF-SB. Stakeholder engagement is a central component of this project via two stakeholder advisory boards that will directly inform and oversee the project.

**Discussion:**

Results of this study will provide evidence about the relative impact of two school-based mental health interventions on outcomes reported as meaningful by caregivers and school providers. The additional focus on evaluating factors that support the implementation of FYF-SB and ZOR will allow future studies to test targeted implementation strategies that support mental health programming uptake and implementation within public schools.

**Trial registration:**

This trial is registered with clinicaltrials.gov (NCT05863520).

**Supplementary Information:**

The online version contains supplementary material available at 10.1186/s12888-023-05441-0.

## Background

As many as 40% of autistic youth develop co-occurring clinical anxiety that can significantly limit their participation and quality of life across multiple contexts [1]. For example, in schools, anxiety can limit students’ attendance, school performance, peer relationships, and extracurricular participation [2–5]. Unfortunately, autistic youth with anxiety have experienced longstanding challenges accessing mental health care in community settings [6, 7]. Difficulties accessing mental health services are even more pronounced for youth from minoritized backgrounds, whose mental health symptoms may go undetected or misinterpreted, thus, limiting their access to care or leading to punishment-based interventions [4]. Youth from minoritized communities were also disproportionately affected by the COVID-19 pandemic, as service access was reduced due to long wait lists and a shortage qualified providers [8, 9]. Without equitable access to evidence-based mental health care, underserved autistic individuals may experience more impairing anxiety over time, further impacting their quality of life [10].

Recent systematic reviews have indicated that cognitive behavioral therapy (CBT) is an evidence-based practice (EBP) that is effective in reducing anxiety symptoms for children with and without autism [11, 12]. However, the vast majority of CBT research for autistic youth has been implemented within university settings and primarily by mental health providers (e.g., psychologists) [12, 13]. Schools hold great promise to equitably manage the mental health symptoms of autistic youth. Approximately 65% of autistic youth receive mental health services within schools [14], and youth with minoritized identities often only access mental health services within school settings [15]. Yet most autism research conducted within schools has focused on the implementation of social communication interventions for young autistic children [16, 17], rather than interventions focused on improving anxiety and other mental health outcomes.

CBT has been successfully implemented in schools for non-autistic youth with anxiety symptoms [18, 19], and a handful of studies have adapted CBT for autistic youth with anxiety for school-based delivery, with encouraging results [20, 21]; however, the long-term viability of such programs is limited, since these studies relied heavily on research personnel to deliver or co-deliver these interventions rather than school-based personnel. Further, in the absence of formal training in CBT, general and special education teachers are less likely to use CBT core components such as encouraging autistic students to face their fears students [22]. To address the significant mental health needs of autistic students, there is a critical need to build the capacity for school-based providers to deliver EBPs without continued support from clinical providers. Importantly, training non-autism experts offers the possibility to dramatically expand capacity to serve autistic youth [16].

Our team has attempted to address this gap through the development of the Facing Your Fears-School Based Program (FYF-SB), an evidence-based group CBT intervention that can be delivered by interdisciplinary school providers (ISPs) to anxious autistic youth [23–25]. FYF-SB was derived from FYF, an empirically supported, clinic-based, outpatient group CBT program for autistic youth ages 8–14 years. FYF-SB was iteratively adapted through extensive caregiver, school provider, and school administrator input that underscored the lack of available and appropriate school-based interventions for anxious autistic youth and necessary adaptations to FYF for school-based delivery [26]. FYF-SB was then piloted by 25 ISPs (e.g., school psychologists, special educators, speech language pathologists) and delivered to 29 students with autism. Results indicated that significant reductions in anxiety occurred following program participation [27]. Following the feasibility trial, 81 students with autism or suspected autism and anxiety were randomized to either FYF-SB or usual care. ISP fidelity to FYF-SB was strong and significant reductions in anxiety were apparent for students receiving FYF-SB relative to students in usual care according to parent- and student-report [28].

Results from these initial trials are promising, yet FYF-SB has not yet been compared to another readily available school mental health program. Indeed, sweeping reviews of CBT effectiveness also indicate that although CBT approaches have consistently demonstrated superiority to wait list or no treatment, there is no clear evidence that CBT is more effective than other interventions for anxiety. The current trial aims to address this gap by comparing FYF-SB to the Zones of Regulation (ZOR) [29], a manualized school-based intervention that does not have an established evidence base [30], yet is in wide-spread use with over 300,000 books sold and thousands of educators having delivered the program [31]. ZOR is also rooted in CBT principles and uses a metacognitive framework to increase emotion regulation skills by increasing awareness of feelings and building regulation, prosocial skills, and overall wellness. Emotion dysregulation is common in autistic youth, is a correlate of anxiety, and is a transdiagnostic factor that underlies various psychiatric conditions, including anxiety, depression, and other mood disturbance [32]. Given its underlying role in mental health conditions, emotion regulation is a critical target of anxiety-based interventions.

Comparing the effectiveness of FYF-SB and ZOR is also important given that ZOR and FYF-SB have related but distinct treatment approaches. That is, although both interventions are rooted in CBT principles, ZOR primarily uses psychoeducation and direct teaching of coping strategies to improve self-regulation, while FYF-SB pairs psychoeducation with graded exposure in which students practice facing their fears a little bit at a time to reduce anxiety symptoms. Although graded exposure plays a critical role in anxiety outcomes and is considered a core component of CBT [33, 34], previous research has demonstrated that graded exposure is often a novel treatment strategy for school-based providers that may be logistically complicated to implement within schools [35]. Thus, in addition to their distinct intervention targets, comparing ZOR and FYF-SB may provide insight into the feasibility of each intervention’s core components when implemented within school settings.

The goal of this protocol paper is to describe a multi-site study comparing two school-based interventions, FYF-SB and ZOR via a cluster randomized controlled type 1 hybrid effectiveness-implementation trial [36] to determine which of the two interventions will best support autistic youth with anxiety in schools. Elementary and middle schools will be randomized to deliver either FYF-SB or ZOR to autistic students with interfering anxiety in small group format*.* Specific aims are as follows: 1) Compare the effectiveness of FYF-SB and ZOR on: (a) symptoms of anxiety (primary) and emotion dysregulation (co-primary) as rated by caregivers, students, teachers, and masked research staff; and (b) functional outcomes of priority to stakeholders, including school attendance, disciplinary action, and academic participation; 2) Compare the acceptability, appropriateness, and feasibility of FYF-SB and ZOR and a range of factors that contribute to the successful implementation of these two programs within schools; and 3) Examine autism severity and anxiety symptom severity as moderators of treatment response.

Results of this study will expand the intervention and implementation literature for autistic youth with co-occurring mental health conditions by providing evidence about the relative impact of two school-based mental health interventions on outcomes reported as meaningful by caregivers and school providers. The additional focus on evaluating factors that support the implementation of FYF-SB and ZOR will allow future studies to test targeted implementation strategies that support mental health programming uptake and implementation within public schools in order to meet the needs of autistic students and their families.

## Methods

### Study setting

The current trial takes place within public school districts and charter schools across two states representing racial, ethnic, economic, and geographic diversity (i.e., Colorado and North Carolina). Participating districts include large, medium, and small districts serving both urban and rural communities. Recruitment will be prioritized to occur within elementary and middle schools with high rates of free and reduced lunch as well as racially and ethnically diverse student populations.

### Stakeholder involvement

Stakeholder engagement is a central component of this project via two stakeholder advisory boards (SABs) that will directly inform and oversee the project. The first SAB is composed of parents of autistic youth, school providers and administrators from participating districts, as well as autistic adults with experience participating in CBT. The second SAB is composed of a minimum of 6 high school autistic students (High School-Stakeholder Advisory Board; HS-SAB) from Colorado and North Carolina. They will be asked to share their lived experience with autism and anxiety in schools and what kinds of support would have been most helpful to them as younger students. The two SABs will meet a minimum of twice yearly and inform all phases of the project, including advise on recruitment strategies, trouble-shoot challenges with conducting the research, review quantitative and qualitative data, and provide recommendations for communicating research findings to study participants. Finally, an autistic adult is a co-investigator on the research team, in a shared decision-making role. She will attend regular research team meetings and co-facilitate both SABs.

### Procedures

School administrators from each partnering school district will first identify elementary and middle schools in their districts that serve a high proportion of historically underserved youth (e.g., racial/ethnic minority youth; high rates of free and reduced lunch). If they are unable to recruit enough participants from those schools, then district leaders will turn to other schools within their district for recruitment. Two-hundred autistic students from up to one hundred (100) schools across Colorado and North Carolina will be randomized to either ZOR or FYF-SB over the course of the project, with up to 50 schools randomized during one academic year and another 50 schools randomized over a second academic year. Once schools are randomized to condition, a minimum of two ISPs at each school will be consented and participate*.* All ISPs will receive training on how to recognize anxiety and emotion dysregulation in autistic students followed by training in either FYF-SB or ZOR.

After training, ISPs will nominate students in their school that they suspect may meet the study inclusion criteria to form groups of 2–5 students with autism and co-occurring anxiety. Once the screening and qualification processes have been completed, and informed consent/assent has been obtained by trained research staff, pre-intervention measures will be completed. Caregiver- and teacher-report measures may be completed electronically via REDCap while student self-report measures will be completed in person by a member of the research team. If students meet inclusion criteria, they will be given the option to assent into the study. Assent will be obtained at their school and by trained research staff. If they assent, participating students will receive either ZOR or FYF-SB depending on which condition their school was randomized to. FYF-SB and ZOR will be delivered over 12 weeks for a duration of approximately 40 min for each lesson. Post-intervention measurements will be completed within 4 weeks of program completion. Semi-structured exit interviews will be conducted with a purposive sample of students, caregivers, and ISPs to obtain information regarding acceptability, appropriateness, and feasibility of either ZOR or FYF-SB, along with any potential harms. Selected outcome measures will be completed at 6 months follow-up.

### Interventions

*Facing Your Fears-School Based* (*FYF-SB)* is derived from FYF, an evidence-based outpatient clinic program for autistic youth (and their caregivers) between the ages of 8–14 years [37]. The original FYF program is a 14 week, 90-min, group CBT intervention focused on the management of clinically significant anxiety symptoms. Seven treatment studies have been conducted on FYF, including a randomized trial, a multi-site randomized trial, and several pilot studies adapting FYF for different populations [23, 24, 27]. FYF-SB was adapted for school-based delivery based on stakeholder input and is a group CBT program [26]. Consistent with the original program, FYF-SB consists of psychoeducation (e.g., identification of anxious symptoms, somatic management strategies, use of positive self-statements) and graded exposure practice (facing fears a little at a time). Worksheets include many visuals, paired with clear written directions, and multiple-choice lists. Brief “hands-on” activities and video modeling enhance accessibility of CBT content for different learners. Culturally appropriate representations of students from diverse racial/ethnic backgrounds are incorporated throughout the materials, and this program has been delivered to students from historically underserved backgrounds. Results from a pilot feasibility study and randomized trial have demonstrated effectiveness of FYF-SB compared to usual school care [27].

*Zones of Regulation (ZOR)* uses a metacognitive framework to increase awareness of feelings, as well as various tools and strategies to improve regulation and build prosocial skills and overall wellness. “Mindfulness, sensory integration, movement, thinking strategies, wellness and healthy connections” are examples of some of the tools and strategies included in ZOR. ZOR is an inclusive strategy for neurodiverse learners who have specific social/emotional and/or behavioral needs [29]. ZOR incorporates Social Thinking concepts to support students to identify feelings, identify level of alertness and understand how their behaviors can impact outcome. ZOR is rooted in CBT principles and supports students to self-monitor their own behaviors and eventually engage in management strategies independently. As mentioned, ZOR is clearly in widespread use and has been delivered by thousands of educators. In this study, ZOR will be delivered in small groups and with similar duration and frequency as FYF-SB*.* See Table 1 for a depiction of the core components of FYF-SB and ZOR.

**Table 1:** Core activities of FYF-SB and ZOR

	**Facing Your Fears**	**Zones of Regulation**
**Psychoeducation**		
Identification and understanding emotions	X	X
**Introduction and practice of coping skills**		
Coping skills specific to anxiety	X	
Teaching cognitive reappraisal	X	
Creation and externalization of "worry bully"	X	
Understanding impact of emotions on others		X
Use of sensory regulation strategies		X
**Exposure**		
Practice facing fears a little at a time	X	
**Generalization**		
Handouts shared with caregivers	X	X
At least one point of contact with caregivers	X	

### Randomization

Schools within each state will be randomized with 1:1 ratio into FYF-SB or ZOR comparators. To minimize the imbalance in participant age, randomization will be stratified by school type (elementary versus middle school). The unit of intervention and randomization will be at the school level in order to feasibly train ISPs for one type of intervention and to prevent contamination of comparators within a school across students.

### Training workshops

Both the ZOR and FYF-SB training workshops will be approximately 12 h in duration. The trainings will be scheduled in collaboration with the participating school districts. Each training will include an initial segment (3-h overview) on how to identify anxiety and emotion dysregulation in students with autism so that ISPs will be best prepared to nominate students for participation in the study. Additional content of the workshops will vary by intervention approach, but will include a conceptual overview of the program, as well as session by session review. Both trainings will include a combination of didactic presentations and discussion. Some trainings will be delivered virtually while others will be delivered in person (per school district choice). Checklists for fidelity to the training will be used to ensure that all trainings are conducted in the same manner.

### Consultation

The ISPs teams will participate in bi-monthly consultation sessions (20–30 min) via phone or video call with a member of the research team, for the duration of the time they deliver the intervention for a maximum of 6 consultation calls (i.e., 3 h) per school team. The consultation format for each call will include opportunities for reflection on group facilitation as well as the following components: (a) informal question and-answer period; (b) provision of feedback regarding strengths of facilitation, missing elements, and suggestions for delivery of session content; and (c) plan for the upcoming sessions. The ZOR consultation will be provided by research team members trained to reliably provide consultation in ZOR. Similarly, the FYF-SB consultation will be provided by trained research personnel.

### Participants

#### Interdisciplinary school providers (ISPs)

Recruitment will include up to 200 ISPs who (1) are degreed professionals in one of the following: education (special or general education), school psychology, counseling, social work, speech/language pathology, occupational or physical therapy; and (2) work with autistic students with anxiety. ISPs must also be able to complete study requirements including attending the training workshops, delivering at least 80% of the program they were randomized to, and participating in 80% of bi-monthly consultation sessions with the research team. At least two ISPs per school team will facilitate the FYF-SB or ZOR groups. It is recommended that the ISP teams have access to ongoing consultation with a school mental health provider. Paraprofessionals can be enrolled as ISPs, provided that their role is to assist other ISPs, rather than lead the groups themselves.

#### Autistic students

Two hundred students ages 8–14 years currently being served on an Individualized Education Plan (IEP) under any educational designation will participate. Recruitment will be prioritized within historically underserved communities given longstanding disparities in access to mental health care. Students will be included if they have: (1) a known medical diagnosis of autism, an educational identification of autism, OR suspected autism; AND (2) clinical anxiety according to either student, parent or teacher report. Students with suspected autism will be included if they have clinically significant impairment (T-score above 70) in reciprocal social behavior according to the Social Responsiveness Scale – Second Edition (SRS-2) [38] or if they exceed the cut-off score on the Social Communication Questionnaire, Lifetime Version (SCQ) [39] or the Autism Diagnostic Observation Schedule, Second Edition (ADOS-2) [40] administered by the research team. Clinical anxiety will be determined by: (1) significant elevation on the Total Score of the parent or child report of the Screen for Child Anxiety and Related Emotional Disorder (SCARED) [41]; (2) meeting or exceeding the cut-off for any of the subscales of the parent or child report of the SCARED; or (3) clinical elevations on the School Anxiety Scale – Teacher Report (> 17; SAS-TR) [42]. Students will be excluded if they have (1) known intellectual disability; (2) significant behavior or psychiatric challenges that prevent them from participation in small group activities; (3) lack of parent or caregiver permission for participation; or if students are: (4) actively receiving CBT targeting anxiety within the community. Students with intellectual disabilities will be excluded from the current trial because the cognitive and linguistic demands of the interventions are not tailored for youth with more significant learning challenges.

#### Caregivers

Two hundred caregivers of students 8–14 years defined above will be included. Participating caregivers will be asked to complete survey measures for their child and the PARS-ASD, participate in one parent contact, and complete surveys, the PARS-ASD, and exit interviews following their child’s participation in either ZOR or FYF-SB.

#### Measures

All measures will occur at baseline, post-treatment, and 6-month follow-up. Semi-structured interviews will also be conducted with a purposive sample of students, caregivers, and school providers. See Table 2 for a timeline of enrollment, study procedures, and measures.

**Table 2:**
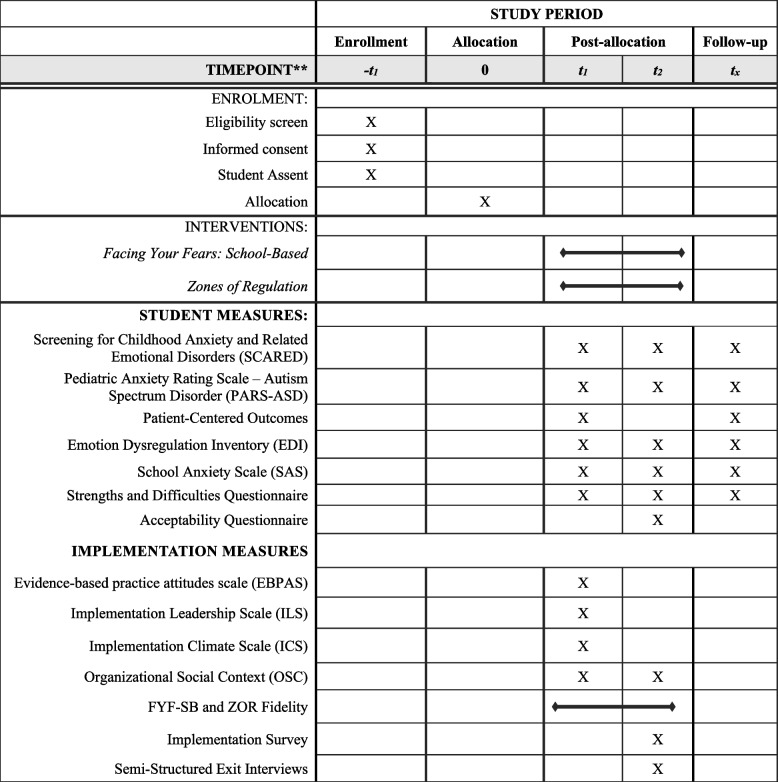
Schedule of enrollment across study time period

^**^t_1:_ Pre-FYF-SB/ZOR; t_2_: Post-FYF-SB/ZOR; t_x_: 6-month follow-up

### Student measures

#### Demographic Information

Caregivers will complete demographic information about their participating child and family. This includes the child’s age, race, ethnicity, grade and school placement, developmental and medical diagnoses, and school and community service history. Caregivers will also provide their own age, gender, race, ethnicity, highest educational obtainment, annual household income, household size, marital status, occupation, and the extent to which they experience psychosocial stressors such as food, housing, transportation, and healthcare related insecurities.

Primary outcome. screening for childhood anxiety and related emotional disorders – child/parent versions (SCARED) [41]*.* The SCARED is a 41-item inventory of statements that assesses five types of anxiety experienced by children and adolescents, to be completed separately by caregivers and students pre/post intervention and at 6 months follow-up. A total score, as well as cutoffs for specific domains of anxiety (e.g., social, generalized) are obtained. A total score of 25 or higher is clinically significant. Youth who obtain a score above the clinical cutoff for the total score or for 1 or more domains are eligible. The SCARED has been used in previous FYF-SB trials as well as in other studies with autistic children [24, 28]. Results from our previous research confirm the 41-item measure’s five-factor structure and suggest good sensitivity (0.71) and specificity (0.67) among parents of autistic youth [28].

Co-primary outcome. pediatric anxiety rating scale-autism spectrum disorder (PARS-ASD) [43]. The PARS-ASD is a clinician-rated semi-structured interview assessing anxiety severity and impairment over the past week, modified for autistic youth ages 5 to 17 from the original Pediatric Anxiety Rating Scale (PARS) [44]. The PARS-ASD will be administered by an independent evaluator (IE) masked to condition. It includes a Symptom Checklist and five Severity Items. The Symptom Checklist covers social anxiety, separation anxiety, generalized anxiety, panic, specific phobia, and other anxiety symptoms. The severity of symptoms is examined by assessing the frequency and pervasiveness of anxiety, as well as the extent to which the child avoids anxiety provoking situations (e.g., behavioral manifestations of anxiety). Interference with family relationships within the home and interference with peer and adult relationships and/or performance outside of the home are also examined. Preliminary psychometrics were strong: internal consistency was 0.90; convergent validity was supported, as the PARS-ASD was strongly correlated with other parent-reported anxiety measures (*rs* = 0.62–0.68); divergent validity was also supported as the PARS-ASD had low correlations with parent ratings of social withdrawal, stereotyped movements, hyperactivity and repetitive behaviors [43].

Stakeholder identified outcomes. In previous research studies, stakeholder partners listed the following as meaningful treatment outcomes within schools: number of tardies, attendance, grades, number of elopements (leaving school area without permission), amount of classroom participation, number of expulsions and suspensions, number of visits to the principal and nurse, use of coping strategies, increase in self-advocacy, number of phone calls home, number of referrals to the social worker, increase in social initiations with others, and decrease in outbursts [26]. We will operationally define these outcomes and partner with school teams and school personnel to collect each of these outcomes on participating students from the academic year prior to study participation and the academic year in which students participated in FYF-SB or ZOR.

Emotion dysregulation inventory (EDI) [45]. The EDI is a parent report measure of emotion dysregulation in autistic youth and will be completed pre/post intervention and at follow-up. The EDI can be used as a self-report measure and can also be completed by school providers. Items are rated on a 5-point Likert scale ranging from 1 (not at all) to 5 (very severe). The Reactivity short form (7 items) and Dysphoria factor (6 items) have demonstrated good internal consistency in autistic youth (Chronbach’s alpha = 0.92 for Reactivity Short Form; Chronbach’s alpha = 0.90 for Dysphoria).

The school anxiety scale, teacher report (SAS-TR) is a 16-item teacher-reported measure of anxiety designed to assess the behavior of children at school from 5 to 12 years of age [42]. Items are answered on a four-point scale. The measure provides a total score for anxiety (scores ranging from 0–48). It includes two subscale scores (reflecting social anxiety and generalized anxiety). The SAS-TR will be administered pre/post intervention and follow-up. This measure has been used in our previous school-based trials as well as in other school-based research with autistic students [21]. Internal consistency for both the total anxiety score (Cronbach’s alpha = 0.93) and the subscales (Cronbach’s alpha = 0.92) were strong. Test–retest reliability (intra-class correlations (ICC) ranged from 0.73-0.81) was also strong. Finally, convergent and discriminant validity were also good (0.76; -0.16; respectively).

Strengths and difficulties questionnaire (SDQ) is a 25-item teacher, parent and student reported tool measuring prosocial behavior and psychopathology of youth 3–16 years old [46]. The SDQ evaluates five-factors: emotional symptoms, conduct problems, hyperactivity/inattention, peer relationship problems, and prosocial behavior. Studies on the psychometrics of the SDQ have yielded satisfactory internal consistency (mean Cronbach’s α: 0.73), cross-informant correlation (mean: 0.34) and retest stability after 4 to 6 months (mean: 0.62). The SDQ has also been successfully used in autism research [47]. It will be administered pre/post intervention and at follow-up.

### Implementation measures

ISP demographic information. ISPs will indicate their age, race, ethnicity, gender identity, and disciplinary background. They will also indicate their years of experience working within their professional role, working with autistic students, and working with autistic students who have co-occurring anxiety.

Evidence-based practices attitudes scale (EBPAS) is a 15-item measure of providers' attitudes towards adopting novel, evidence-based practices [48]. It contains the following subscales: Requirements; Appeal; Openness; Divergence. Providers will rate the extent to which they agree with statements using a 5-point Likert scale with a 0 indicating “not at all,” and a 4 indicating, “to a very great extent.”

School Provider Stress and Burnout. ISPs will complete the 5-item Emotional Exhaustion subscale of the Organizational Social Context Questionnaire [49] to capture the fatigue and stress dimension of burnout. Each ISP will report their level of agreement on a 5-point Likert-type scale (1 = Strongly disagree to 5 = Strongly agree) to statements about their sense of emotional exhaustion from their work (e.g., “I feel burned out from my work.”).

Implementation leadership. ISPs will rate the extent to which their school supervisors are providing leadership in the use of EBPs using the 12-item Implementation Leadership Scale (ILS) [50]. The ILS assesses a leader’s behavior with regard to EBP implementation across four main domains: proactive, knowledgeable, supportive, and perseverant. ISPs will rate each item using a 5-point Likert scale with a 0 indicating “not at all” and 4 a “very great extent.” Past research has demonstrated that the ILS has excellent internal consistency, as well as convergent and discriminant validity (Aarons et al., 2014). It has also been used in other school studies for autism with strong internal reliability (Williams et al., 2021).

Implementation climate. ISPs will rate their school’s evidence-based practice (EBP) implementation climate using the Implementation Climate Scale (ICS) [51]. The ICS includes 18 items rated on a Likert scale from 0 (not at all) to 4 (very great extent). Items are averaged to produce a total score. Item content covers six domains, including focus on EBPs, educational support for EBPs, recognition for EBPs, rewards for EBPs, selection for EBPs, and selection for openness. The scale is psychometrically validated and has been used in other studies examining the implementation of autism EBPs within schools [51, 52].

Intervention fidelity*.* Two separate but similar treatment fidelity checklists will be used to document treatment adherence as well as provider competence in delivering core components of FYF-SB or ZOR. The measures will assess the ISPs’ adherence to their assigned intervention by assessing the presence/absence of core components as well as the quality of intervention delivery. In ZOR*,* the checklist will measure ISPs’ adherence to the four key elements of preparation, provision of structure, quality of facilitation, and adult evaluation of student learning. In FYF-SB, the checklist will measure ISP’s adherence to core activities included within each session. It will also include an overall competence rating in which ISP’s Both FYF-SB and ZOR fidelity will be coded by trained research team members with reliability calculated on 15–20% of scored sessions.

Implementation survey. At the conclusion of delivering FYF-SB or ZOR, ISPs will rate the feasibility, acceptability, and appropriateness of their respective intervention, including the likelihood that providers will continue to implement the intervention once the study has ended. Providers will rate each of 36-items using a 5-point Likert scale. This survey was adapted from several different sources [53] and is designed to examine intervention feasibility, acceptability, and appropriateness for the school setting. An adapted version of this survey has been used in other studies examining the feasibility of interventions for autistic youth, including in our previous school trials [27, 28].

Acceptability questionnaires. A six-item acceptability questionnaire will be completed by participating students asking them to respond to questions such as “How much did you enjoy participating in FYF-SB/ZOR?” or “Do you feel better after participating in FYF-SB/ZOR?” Students will rate each item using a 5-point Likert scale.

Semi-structured exit interviews. A random subset of students, caregivers, and ISPs will be engaged in semi-structured exit interviews conducted by an independent member of the research team. For ISPs, the exit interviews are designed to understand the acceptability, appropriateness, and feasibility of FYF-SB and ZOR, as well as the implementation outcomes of the intervention in which they were trained*.* Interview questions for ISPs will include: 1) overall impressions of the intervention; 2) relative advantage of FYF-SB or ZOR; 3) the feasibility of implementing the intervention; 4) adaptations necessary to support intervention implementation; 5) the perceived impact of the intervention on anxiety outcomes; and 6) the extent to which the intervention will be used in the future. For caregivers and students, interview questions include: 1) overall impressions of FYF-SB or ZOR; 2) the acceptability of the intervention; 3) how FYF-SB or ZOR compared to other mental health interventions received; 4) the impact of FYF-SB or ZOR on anxiety and emotion regulation outcomes; 5) the extent to which caregivers were involved in the intervention and saw skill generalization at home; and 6) whether the caregiver or student would recommend the intervention to others. All interviews will be audio-recorded, transcribed verbatim and de-identified.

### Analytic plan

Between-arm imbalance of baseline characteristics will be examined using* t* and chi-square tests and evaluated based on summary statistics. Intervention effect of FYF-SB as compared to ZOR will be assessed using Linear mixed effect model (LMM) for continuous outcomes and non-linear mixed effect model (NLMM) for categorical outcome such as school attendance. To account for the design effect of clustering trials, random school (cluster) effect will be in the models for outcome without repeated measures while appropriate covariance structure of LMM/NLMM accounting for both the design effect and the correlation of repeated measures will be used for outcome with repeated measures. Transformation of outcome (i.e., Square root or log) will be used if normality assumption is violated. Any imbalanced potential confounding variables will be adjusted for modeling. Primary analysis will be conducted on intent-to-treat basis (ITT). LMM and NLMM will be the primary method for dealing with missing values without data imputation since missing patterns would most likely be of the missing at random (MAR) pattern. The change score from baseline to the end of intervention is considered to the primary effectiveness endpoint. No type I error adjustment for multiple tests in a single model or multiple outcomes will be applied. *P* < 0.05 is deemed statistically significant. No interim analysis will be performed. To examine the robustness of primary analyses, sensitivity analyses will be conducted, including the same analyses among completers and analyses after missing data are imputed using Markov chain Monte Carlo method.

#### Aim 1

The SCARED and PRAS-ASD are the co-primary outcome variables. Both primary and secondary outcomes will be analyzed using the above discussed LMM/NLMM model with fixed effects consisting of time (i.e., baseline/end of treatment/6 months follow-up), comparator (FYF-SB or ZOR) and their interaction. Intervention effect of FYF-SB as compared to ZOR will be estimated by between-comparator difference in the change score of outcome measure from baseline (i.e., interaction) with 95% confidence interval and be tested statistically under the statistical model.

#### Aim 2

Quantitative data will be analyzed in the same fashion as for the outcome without repeated measures in Aim 1. Consistent with CORE-Q reporting guidelines [54], all interviews will be audio-recorded, and transcribed verbatim. ​​Exit interview data will be formally analyzed using conventional content analysis [55, 56]. This form of analysis is commonly used in studies with the aim of describing a phenomenon at a level that closely reflects the transcript content. In the case of Aim 2 data, the content analysis will be used to closely describe stakeholder perspectives of the feasibility (i.e., ease of use), acceptability, and appropriateness of FYF-SB or ZOR, as well as the infrastructure necessary to implement and sustain these programs. All coding will be facilitated by MAXQDA software. Additionally, all qualitative results will be member checked with our stakeholder advisory board members prior to synthesis into various products.

#### Aim 3

To explore the impact of baseline symptoms and other demographic variables on the intervention effect, we will include similar models to those being tested in Aim 1, with the use of an interaction term (i.e., intervention x moderator x time interaction) in addition to related two-way interaction terms. Candidate moderators, including age, will be examined. If an interaction results in significance, we will further test treatment effects within subgroups analogous to hypotheses in Aim 1. Similar analysis will also be conducted with the outcome dichotomized as responder and non-responder using clinical criteria.

### Sample size and power analysis

This study was primarily powered on detecting a between-comparator difference in the primary outcome (i.e., SCARED score) using ITT analysis. We expect the mean cluster size across the 100 schools to be 2 participants, ranging 2–5. Based on the FYF-SB RCT [28], estimates of ICC ranging from 0.001–0.15 were conservatively used in this power analysis. Our study will have 80% power at 5% significance level, with 100 participants per arm to detect a medium effect size (0.45 of Cohen’s D). Additionally, studies with autistic youth that each compared two active treatments further supported our anticipated effect size and power. Two studies reported significant treatment effects when comparing CBT adapted for autistic youth to other interventions (Standard CBT; Enhanced Standard Community Treatment) [57, 58]. The effect size when comparing these two active comparators was medium in size (ranging from 0.50- 0.63) [57, 58]. The effect sizes from these two studies are nearly equivalent to the effect size used for our current study, given that all are within the medium category according to effect size interpretation [59]. We will also have sufficient power to detect the medium effect size for the secondary outcomes.

### Trial status

All universities and, when possible, school district Institutional Review Boards have approved the study procedures. This trial is also registered with clinicaltrials.gov (NCT05863520; May 18, 2023). At the time of submission of this manuscript (Fall 2023), we have enrolled participants (e.g., interdisciplinary school providers, autistic students) from elementary and middle schools for data collection.

## Discussion

Anxiety is common amongst autistic youth [60] and can significantly limit their full participation across home, community, and school settings [61]. In school, anxiety may negatively impact students’ attendance, school performance, peer relationships, and extracurricular participation [4]. Although CBT is the gold standard treatment for anxiety in autistic youth, accessing evidence-based mental health care in community settings is difficult for autistic youth and their families [6, 7]. Schools may be an ideal location to address this gap and to equitably manage the mental health symptoms of autistic youth, yet evidence-based practices such as CBT have not been consistently available in schools. Further, previous work with school administrators, providers, and caregivers has clearly indicated that these partners recognize the significant anxiety that many autistic students experience, yet do not have evidence regarding which CBT programs may be most beneficial to implement within school settings to serve autistic youth more equitably and that address outcomes that are meaningful to autistic students and their families [4, 26].

This trial will address this gap by comparing the effectiveness and implementation outcomes of two CBT programs for autistic students across elementary and middle schools. The two interventions that are the focus of this trial*,* FYF-SB and ZOR, are rooted in CBT principles but manage anxiety in different ways. FYF-SB incorporates psychoeducation and graded exposure (facing fears a little at a time) to specifically target anxiety symptom reduction. On the other hand, ZOR targets emotion dysregulation which often underlies anxiety symptoms, and uses predominantly psychoeducation to teach students strategies to regulate different emotional states. The current trial will also address important implementation questions by also comparing the feasibility, acceptability, and appropriateness of each intervention according to students, caregivers, and school providers, and by examining factors that support and hinder the implementation of each program within school settings.

The development and implementation of this trial was directly informed by autistic students and adults, caregivers, school providers, and administrators. Continued partnership with two advisory boards throughout the trial will help ensure the meaningful engagement of autistic and school partners, the measurement of patient-centered outcomes, and the dissemination of practical information about the strengths and weaknesses of FYF-SB and ZOR so that school leaders and other stakeholders can make informed decisions about program selection to support autistic students.

### Limitations

This is one of the first studies to directly compare the effectiveness and implementation outcomes of two school-based CBT programs for autistic students with anxiety. The cluster randomized trial is designed to use methods that are both rigorous (e.g., the use of masked evaluators for primary outcomes) yet that are also pragmatic within school settings. For example, rather than confirming autism diagnoses using resource-intensive, standardized assessments, this study will include students with an educational identification of autism or who have significantly elevated social communication differences as indicated by one or more screening tools. Thus, it is possible that students will be included who are not autistic. However, this more pragmatic inclusion criteria reduces measurement burden on students and caregivers and likely ensures a sample of students that is more reflective of students with social communication differences being services in schools. In addition to the use of pragmatic measures, this study intends to ensure that ZOR and FYF-SB training procedures are similar in length and format. However, it is possible that that attempting to standardize training and consultation masks meaningful differences in how these two programs are implemented in schools, and/or that some of the continued differences methods may impact how the two programs are implemented. Thus, there will be careful documentation of training procedures and the use of mixed methods to understand the role of training content and format on the ease of implementing the two programs.

### Supplementary Information

**Additional file 1.**

## Data Availability

The application described in this manuscript is freely available. Please contact the senior author (JR) for more information.

## Abbreviations

ADOS-2: Autism Diagnostic Observation Schedule
CBT: Cognitive Behavioral Therapy
EDI: Emotion Dysregulation Inventory
EBP: Evidence-Based practice
EBPAS: Evidence-Based Practices Attitudes Scale
FYF-SB: Facing Your Fears, School-Based
ICC: Intraclass Correlation
ICS: Implementation Climate Scale
ILS: Implementation Leadership Scale
ISP: Interdisciplinary School Provider
ITT: Intent to Treat
LMM: Linear mixed effect model
MAR: Missing at random
NLMM: Non-linear mixed effect model
PARS-ASD: Pediatric Anxiety Rating Scale—Autism Spectrum Disorder
SAB: Stakeholder Advisory Board
SAS-TR: School Anxiety Scale – Teacher Report
SCARED: Screen for Child Anxiety and Related Disorders
SCQ: Social Communication Questionnaire
SRS-2: Social Responsiveness Scale, Second Edition
ZOR: Zones of Regulation
